# Updated Freudian drive theory: childhood trauma, epigenetics, serotonin and beta endorphin

**DOI:** 10.3389/fpsyt.2026.1827617

**Published:** 2026-07-06

**Authors:** Michael Kirsch

**Affiliations:** Medical Faculty of the University of Cologne, Department of Anesthesiology and Intensive Care Medicine, University Hospital of Cologne, Cologne, Germany

**Keywords:** attachment dysregulation, Freudian drives, mu-opioid signalling, trauma-related depression, β-endorphin

## Abstract

Sigmund Freud developed Drive Theory from clinical observations and proposed that a limited set of basic drives, including hunger, thirst, sleep, sexuality, and attachment, contributes to psychological stability. Modern reinterpretations allow these drives to be discussed alongside neurobiological regulation, including serotonergic and opioidergic signalling, which are relevant to depressive disorders. Selective serotonin reuptake inhibitors remain a common first line pharmacological option, yet treatment response varies, and simplified deficiency accounts are debated in the current literature. Early adversity is a transdiagnostic risk factor for affective disorders and may contribute to a subtype of depression in which attachment disturbance is prominent. In this context, trauma related epigenetic modifications have been reported in genes involved in serotonergic signalling and related pathways. Reduced serotonergic regulation may be linked to diminished β-endorphin tone, a mechanism discussed in clinical and translational work. Such opioid hypoactivity may also help to frame comorbid behaviours, including overeating and substance use, as short term compensatory attempts rather than independent primary disorders. This manuscript proposes a revised Freudian Drive Theory to describe trauma related depression as a disorder of impaired attachment related motivation with downstream effects on β-endorphin mediated satisfaction. The model is intended to provide a clinically oriented explanatory model. It generates testable predictions and may help to clarify why pharmacological response differs across trauma related presentations, while emphasizing the complementary role of psychotherapy in addressing attachment difficulties, interpersonal functioning, and emotion regulation.

## Introduction

Depression is a widespread and debilitating psychiatric disorder characterised by persistent low mood, reduced pleasure, and cognitive and physical symptoms that compromise daily functioning (1). Its origins are multifactorial, involving biological, psychological, genetic, and environmental influences. Symptoms may include exhaustion, impaired concentration, altered sleep or appetite, and pervasive guilt or worthlessness (2).

For many years, biological models of depression have been discussed within a broader monoaminergic framework (3). Serotonin has been a prominent component of this literature, partly because selective serotonin reuptake inhibitors became a standard first line pharmacological option. Earlier accounts were sometimes simplified into a deficiency narrative. This remains clinically familiar, but it does not capture current evidence well. Large scale reviews have not consistently supported a straightforward serotonin deficiency model (3). The interpretation of these reviews has also been contested, including detailed methodological critiques and rebuttals (4, 5). Moreover, response patterns to selective serotonin reuptake inhibitors remain heterogeneous. Up to half of patients do not achieve remission following initial treatment (6). The delayed onset of improvement further suggests adaptive or downstream mechanisms beyond acute serotonergic modulation (7). These limitations motivate broader explanatory models.

In response, alternative neurochemical hypotheses have been proposed. One influential concept is the endorphin deficiency hypothesis, which suggests that reduced endogenous opioid activity, particularly diminished β-endorphin signalling, contributes to core depressive symptoms such as anhedonia and emotional blunting (8, 9). β-Endorphin is synthesised in the hypothalamus, anterior pituitary, and brainstem. Through its action on mu opioid receptors, it modulates mood, stress responsiveness, and aspects of emotional regulation (9–11). Altered β-endorphin activity has been reported in major depressive disorder, post traumatic stress disorder, and related affective conditions (12, 13).

Findings vary, but multiple studies document altered β-endorphin function in depression (14–19), including low baseline levels or blunted stress induced release (8). Several antidepressant interventions, including selective serotonin reuptake inhibitors, electroconvulsive therapy, and physical exercise, as well as the comorbidity of obesity, are associated with increased β-endorphin concentrations (11, 20). These associations are clinically relevant, although causal direction remains difficult to establish.

Bidirectional associations between depression and comorbid conditions such as addiction (16) complicate causal interpretation. In this manuscript, childhood trauma is not treated as a necessary or sufficient cause of depression. It is regarded as a transdiagnostic risk factor that can increase vulnerability to affective disorders. The present model focuses on a specific subtype in which early trauma and attachment disturbance are prominent. While depression may intensify recollection of adverse events, it does not retroactively produce trauma. Identifying biochemical pathways through which early trauma exerts its effects remains important for treatment, particularly when standard pharmacological approaches show limited benefit. Clinicians currently lack a unified framework that helps to anticipate when selective serotonin reuptake inhibitor treatment is likely to be effective.

This manuscript proposes a revised Freudian Drive Theory to explain how childhood trauma related depression, here termed childhood trauma related depression (CTDD), may arise and lead to additional comorbidities. The model assumes that early trauma can induce epigenetic changes that alter activity within key components of the attachment drive, with downstream effects on β-endorphin tone. Depression related behaviours may emerge directly from this deficit or function as compensatory attempts to mitigate it. Because predictions of the model align with several clinical observations, the updated drive model may aid therapeutic decision making, while remaining open to empirical testing.

## Freudian drive theory

### Traditional Freudian drive theory

Freud developed his drive theory in an attempt to explain psychopathological phenomena observed in patients suffering from neurosis, depression, and psychosis. He distinguished between life-preserving drives (*Eros*), which included hunger, thirst, sexuality, and the need for attachment, and so-called “death drives” which he associated with self-destructive and addiction-like behaviours such as masochism (20).

**Figure 1:**
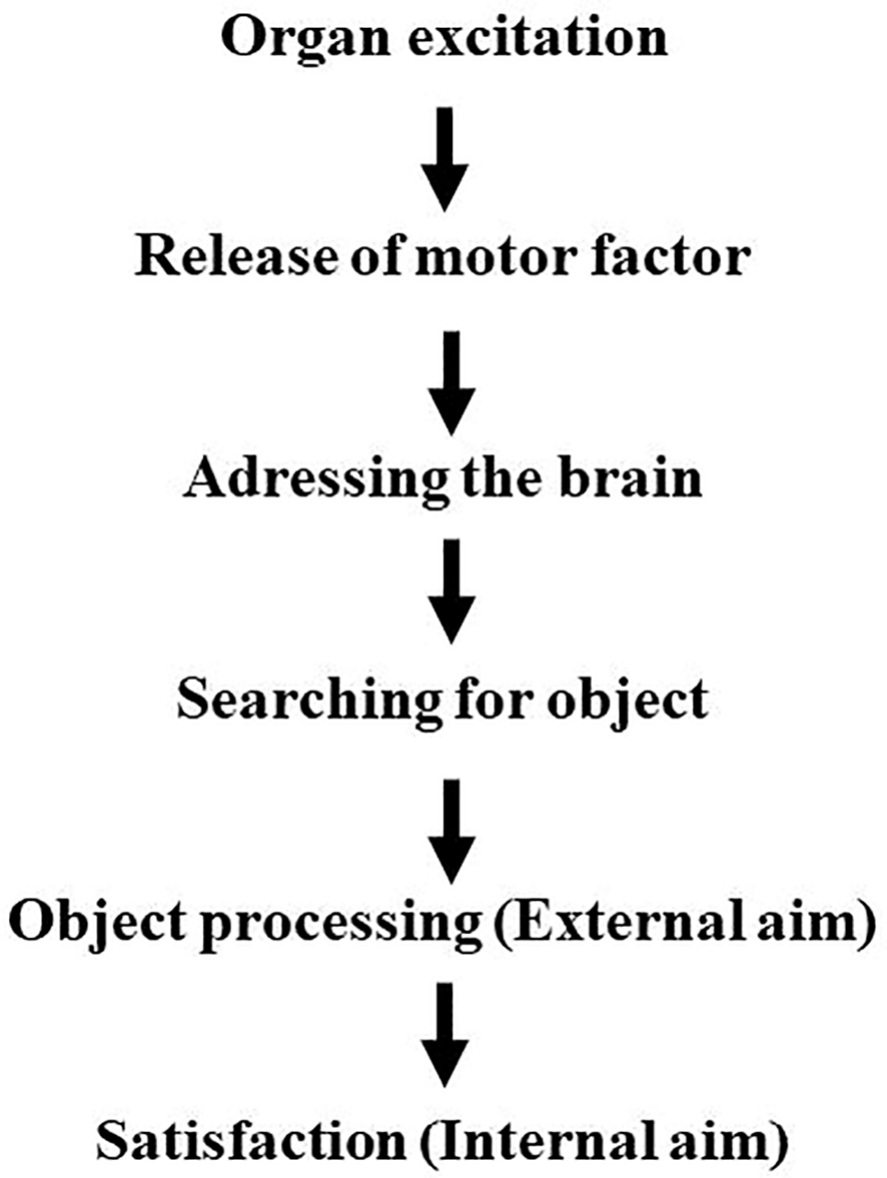
Classical architecture of Freudian drives (1905–1933). Modified from Kirsch, 2025 (34).

Between 1905 and 1933 Freud repeatedly revised this framework (21–23) (**Figure 1**).

Freud’s theoretical work was constrained by the scientific knowledge of his time. Neurochemical concepts such as neurotransmitters, hormones, or endogenous opioids were not yet available. As a result, he relied on abstract constructs to describe processes that are now accessible to biological investigation. Terms such as “motor factor” or “internal aim” were therefore conceptual placeholders rather than mechanistic explanations.

Despite these limitations, Freud emphasised that the number of drives should remain limited and that all drives share a common functional logic. This assumption becomes relevant when evaluating later tendencies to postulate aggression or destructiveness as independent drives, rather than as consequences of dysregulated motivational systems.

### Advances in Freudian drive theory

Later work allows a cautious reinterpretation of Freudian drives in neurobiological terms. A key conceptual advance was provided by Henry, who proposed that chronic hypoactivity of the endogenous opioid system generates strong motivational states aimed at restoring opioid tone (24). Although developed independently of psychoanalysis, this proposal closely parallels Freud’s concept of a shared aim across all drives and implies that drive function contributes to endogenous opioid regulation. β-Endorphin and related peptides induce subjective well-being through mu-opioid receptor activation (10, 11). From this perspective, Freud’s internal aim can be reinterpreted as endogenous β-endorphin release (20, 25).

Subsequent studies suggest that individuals maintain relatively stable levels of central opioid activity (26, 27). Deviations from these levels, whether excessive or deficient, are associated with psychopathological states. Persistently elevated opioid tone has been reported in certain neurodevelopmental conditions, while reduced opioid activity has been described in affective disorders, including bipolar and unipolar depression (16).

### Updated drive model

Integrating these observations permits formulation of an updated drive model that remains conceptually compatible with Freud’s original framework (24, 28–35). In this model, serotonin occupies a key regulatory position, but it should not be treated as a simple mood transmitter. Serotonergic activity contributes to defensive responding, energetic maintenance, arousal regulation, and adaptive behavioural control across brain-body states (36–38). Impairment of serotonergic signalling may therefore affect several drives at once.

**Figure 2:**
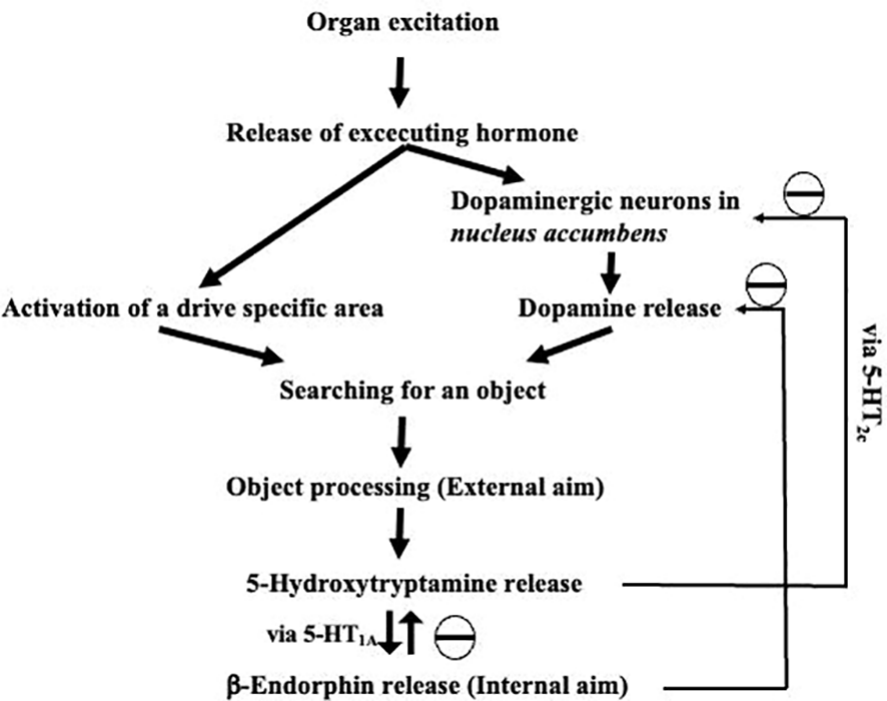
Updated drive architecture (incorporating 1982–2025 findings). Modified from Kirsch, 2025 (34).

**Figure 3:**
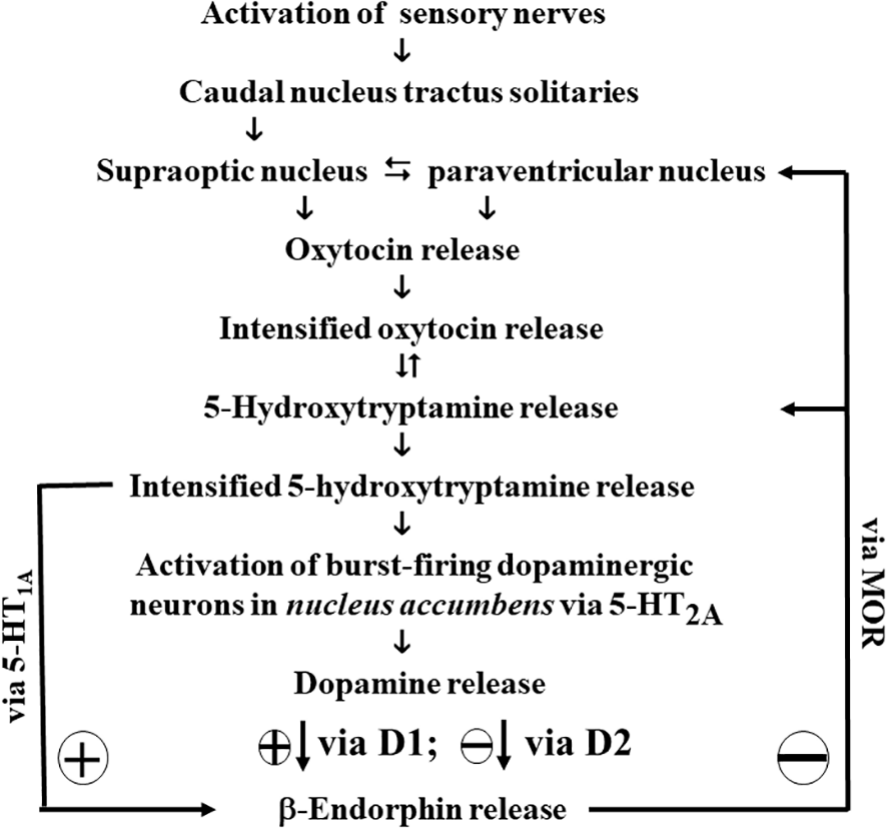
Architecture of the mother-infant attachment system [infant perspective (25, 34)]. Reprinted from Kirsch, 2025 (34).

**Figure 2** presents an updated drive architecture that integrates Freud’s original framework with contemporary neurobiological findings. Core physiological drives (hunger, thirst, sleep, sexuality, see **Figure 2**) and the attachment drive (see **Figure 3**) are retained as distinct motivational systems. **Figure 2** highlights serotonin as a central regulatory element that modulates drive execution and facilitates β-endorphin release via 5-HT_1A_ receptor activation (25, 31–35).

This serotonergic position requires further qualification. Serotonin modulates dopaminergic neurons through several receptor families and in a region-dependent manner (39, 40). The functional meaning of 5-HT_1A_, 5-HT_2A_, and 5-HT_2C_ activity is therefore not identical. In particular, 5-HT_2C_ receptors can exert tonic inhibitory control over dopaminergic and noradrenergic output, whereas 5-HT_2A_ activation may facilitate cortical dopamine release and plasticity under some conditions (41–45). This helps to explain why serotonin can stabilise drive execution in one context, but alter salience, plasticity, or arousal in another.

Earlier publications examined specific organs, hormones, and brain areas engaged by various drives (32). For clarity, **Figure 2** emphasises the central role of dopamine, serotonin, and β-endorphin in drive function. In this model, β-endorphin represents the biological correlate of Freud’s internal aim. It mediates subjective satisfaction and contributes to the termination of motivational pressure.

Dopamine has a different role. It supports learning, salience, approach behaviour, and searching, but it does not provide final satisfaction by itself (29, 30, 35, 46). A tonic/phasic distinction is useful here. Phasic dopamine activity supports cue-driven learning and active seeking, whereas tonic dopamine shapes background responsivity and can constrain subsequent phasic responses (47, 48). This distinction is compatible with the separation between general drive activity in **Figure 2** and attachment-related social responsiveness in **Figure 3**.

**Figure 3** focuses on the attachment drive from the infant’s perspective. It depicts the interaction between serotonergic, oxytocinergic, dopaminergic, and opioidergic systems in the formation of early attachment bonds (25, 34). The attachment drive is not presented as a simple extension of the general drive scheme. It is more dependent on social interaction, tactile input, oxytocinergic regulation, and phasic dopaminergic responses during caregiver contact.

The interaction between serotonin and β-endorphin should not be understood as a simple one way chain. Preclinical microdialysis studies indicate that serotonin can increase extracellular β-endorphin in the *arcuate nucleus* and *nucleus accumbens* (49). In a rat model of depression, this serotonin-induced β-endorphin response was impaired (50). Clinical data also suggest that 5-HT_1A_ receptor related β-endorphin responses may be relevant for antidepressant outcome (51). These findings support a serotonin-to-opioid pathway, but only in a limited and region-specific sense.

The opposite direction is also possible. Endogenous opioids can modulate serotonergic activity and may inhibit serotonin release under certain conditions (52). **Figure 3** should therefore be read as a regulatory loop rather than as a linear cascade. In this loop, serotonergic, oxytocinergic, dopaminergic, and opioidergic systems influence each other according to behavioural context and brain region.

This point is relevant for attachment. Oxytocin and endogenous opioids are both involved in social affiliation, stress buffering, and attachment-related regulation (53–55). Yet the evidence is not equally strong. A recent systematic review found clearer support for an association between oxytocin function and attachment style, whereas opioid findings remain limited by the small number and heterogeneity of available studies (56). The opioid part of the model should therefore be regarded as plausible and clinically relevant, but still in need of more direct empirical testing.

The figure illustrates how successful attachment interactions may support adequate β-endorphin signalling, emotional security, and stress regulation. Conversely, early disruption of caregiving relationships may impair this system and contribute to persistent opioid dysregulation. This interpretation provides a basis for understanding how early trauma can compromise attachment-related motivation and increase vulnerability to later depression.

Importantly, this model (**Figures 2**, **3**) does not treat serotonin as the sole cause of depression. Instead, serotonergic dysregulation is viewed as one regulatory disturbance within a wider motivational network. Dopamine is depicted as a modulatory system for salience, learning, and approach behaviour, while β-endorphin and mu-opioid signalling are more closely related to soothing, reward value, and satisfaction.

Within this framework, aggression and self-destructive behaviours are not conceptualized as independent drives. Rather, they are understood as secondary phenomena that may emerge when core motivational systems fail to restore opioid balance. This interpretation avoids the need to postulate additional drives and remains consistent with Freud’s insistence on a limited drive architecture.

## Childhood trauma, epigenetics, and attachment

### Childhood trauma and attachment disturbance

Attachment theory provides a developmental bridge between early caregiving and later affective regulation (57). Early-life trauma is strongly associated with disturbances of attachment development. Severe neglect, abuse, or inconsistent caregiving increase the risk of long-term impairments in social relatedness and emotional regulation (58–60). In DSM-5, reactive attachment disorder (313.89; F94.1) and disinhibited social engagement disorder (313.89; F94.2) are classified in the chapter on trauma- and stressor-related disorders (1). Both conditions reflect profound disruption of the attachment drive rather than isolated behavioural abnormalities.

**Table 1** summarises prevalence rates of reactive attachment disorder (RAD) and disinhibited social engagement disorder (DSED) across different populations. The data illustrate that severe and chronic early adversity, particularly institutional care and unstable caregiving environments, is strongly associated with attachment pathology (58–60).

**Table 1:** Prevalence and mechanisms of trauma-related attachment disorders.

Population	RAD prevalence	DSED prevalence	Key findings
Institutionalized children	5-22%	10-36%	Highest rates among children raised in orphanages or state care (58, 59)
Foster care children	~5-10%	~10-15%	Rates vary based on stability and age of placement (59)
General population (with maltreatment)	<1-2%	~1-2%	Rare but present in cases of chronic neglect or abuse (60)

Physical contact is a central part of early attachment regulation and should not be reduced to a behavioural detail. In mammalian attachment, tactile stimulation is closely linked to oxytocinergic and opioidergic activity (61). Maternal care also recruits oxytocin, dopamine, and reward-related circuits, which are relevant for both infant regulation and caregiver motivation (62, 63). In humans, oxytocin contributes to social affiliation across parental, romantic, and filial bonds (64). Affectionate caregiver touch may therefore represent one pathway through which attachment experiences are translated into neurobiological regulation (65).

Human work on affective touch further supports this interpretation. Social touch can function as a stress buffer and bonding signal (66). Experimental data also suggest that touch-induced social reward may involve genetic variation in opioid, rather than oxytocin, receptors (67). This point is important for the present model. It suggests that the attachment drive cannot be explained by oxytocin alone. Oxytocin and opioid systems interact, but they may contribute different components to social soothing, reward value, and distress regulation (68).

The mu-opioid receptor system is particularly relevant here. Variation in *OPRM1* has been associated with dispositional and neural sensitivity to social rejection (69). PET data further indicate that the human mu-opioid system responds to both social rejection and social acceptance (70). These findings support the idea that social loss, social safety, and attachment-related comfort recruit opioid mechanisms. They also caution against interpreting β-endorphin tone as a simple peptide concentration. Receptor availability and receptor responsivity matter.

Oxytocin-related findings also require caution. Oxytocin effects depend on sex, hormonal status, receptor variation, and early attachment experience (71). This is consistent with clinical and experimental evidence that oxytocin does not act as a uniform prosocial signal. Its effects depend on context and prior adversity. For this reason, oxytocin is treated here as a component of attachment-related regulation, not as a single marker of secure attachment.

The prevalence data in **Table 1** also show that early adversity does not lead to attachment disorder in every child. This variability is clinically important. Children exposed to institutional or maltreating environments differ in age at exposure, duration of neglect, caregiver availability, genetic background, and later corrective relationships. Studies on institutional care and maltreatment indicate that resilience is not simply the absence of biological change. Rather, some individuals show compensatory developmental patterns that may reduce psychiatric vulnerability despite early adversity (72–74). The present model therefore does not assume a uniform consequence of childhood trauma. It describes one plausible pathway in which early attachment disruption increases vulnerability to later depressive symptoms.

From a developmental perspective, attachment represents one of the earliest and most essential motivational systems. In infancy, attachment-related behaviours ensure proximity to caregivers and facilitate emotional regulation. Successful attachment interactions are associated with adequate serotonergic activity and β-endorphin release, promoting a sense of safety and stress reduction (24, 25, 34, 49, 61–68). When caregiving is unreliable or threatening, this system may fail to establish stable regulation.

### Childhood trauma-dependent epigenetic mechanisms

Trauma occurring during early development can induce long-lasting epigenetic modifications. These include DNA methylation, histone modifications, and changes in non-coding RNA expression, which collectively alter gene expression without changing the underlying DNA sequence (75). Such alterations are relevant for the present model, but they should not be read as deterministic trauma marks.

The stress system should be considered first. Childhood abuse has been associated with increased methylation of the hippocampal glucocorticoid receptor gene promoter *NR3C1*, with reduced glucocorticoid receptor expression in post-mortem brain tissue (76). Early adversity can also interact with *FKBP5* risk alleles, leading to long-lasting changes in glucocorticoid receptor sensitivity and stress-system feedback (77). These findings provide an important bridge between early trauma, HPA-axis regulation, and later affective vulnerability.

The endogenous opioid system interacts with this stress circuitry. Early trauma activates the hypothalamic-pituitary-adrenal axis, and corticotropin-releasing hormone is one of the central mediators of this response. Endogenous opioids can oppose CRH-related stress responses, particularly within noradrenergic circuits such as the *locus coeruleus*, but this regulation may become maladaptive when stress exposure is chronic or repeated (78, 79). In this context, β-endorphin is not simply a marker of well-being. It is part of a wider stress-regulatory system.

The kappa opioid receptor system adds a second, clinically important layer. Dynorphin and kappa opioid receptor signalling are closely linked to dysphoria, stress-induced drug seeking, and relapse vulnerability (80–82). This is relevant for the present model because trauma-related depression may involve not only reduced mu-opioid mediated satisfaction, but also increased aversive opioid-stress signalling. The present model therefore focuses on β-endorphin and mu-opioid tone, but it should be read as part of a broader opioid-stress system rather than as an isolated pathway.

Human studies on childhood maltreatment and DNA methylation remain heterogeneous. Many are cross-sectional, use peripheral tissue, rely on retrospective adversity measures, and focus on candidate genes (83, 84). Genome-wide studies nevertheless support the broader point that childhood abuse can be associated with persistent DNA methylation differences in adulthood (85, 86). The evidence is therefore suggestive, but uneven. This matters for the model.

Several studies describe trauma-associated epigenetic changes in genes involved in serotonergic, oxytocinergic, dopaminergic, and opioidergic signalling. Promoter methylation of the serotonin transporter gene *SLC6A4* has been linked to family history of child abuse and may alter serotonergic regulation (87). For *TPH2*, the rate-limiting enzyme of central serotonin synthesis, early-life stress has been associated with methylation changes and short-term antidepressant response (88). Alterations in *MAOA* methylation may further modulate monoamine degradation in a sex- and genotype-dependent manner (89, 90).

Evidence for *HTR1A* and *HTR2A* is mechanistically relevant, but less specific to childhood trauma (91, 92). These receptors are therefore retained as candidate components of the model rather than as established trauma markers. This distinction is important. Serotonin receptors are widely distributed and involved in many forms of psychopathology.

Epigenetic and molecular findings have also been reported for systems more directly related to attachment, reward, and social stress. Low maternal care has been associated with increased methylation of *OXTR* in peripheral blood cells (93). Other *OXTR* methylation findings support a role in social anxiety, attachment-related regulation, and maltreatment-associated social functioning (94–96). *OPRM1* methylation has been linked to early-life adversity and social distress responses (97), while altered mu opioid receptor availability has been reported in subclinical depression and anxiety (98). Dopamine-related findings, particularly for *DRD2*, support the relevance of reward and motivational salience, although evidence for *DRD1* remains more indirect (99, 100). POMC methylation is included because it may influence endogenous β-endorphin regulation, but its trauma-specific evidence remains limited (101).

**Table 2** is therefore presented as a map of plausible molecular entry points into the attachment drive. It is not presented as proof that each gene alteration has the same clinical weight. Nor does it claim that each alteration independently causes depression.

**Table 2:** Candidate molecular alterations relevant to the attachment drive.

Gene/system	Function	Reported epigenetic or molecular finding	Relevance for the present model	Evidence status	Key refs.
*NR3C1*	Glucocorti-coid receptor	Childhood abuse has been associated with increased *NR3C1* promoter methylation and reduced receptor expression in hippocampal tissue	Links early adversity to altered HPA-axis feedback and stress regulation	Direct childhood abuse evidence; post-mortem brain data	(76)
*FKBP5*	Glucocorti-coid receptor co-chaperone	Childhood trauma can interact with *FKBP5* risk alleles and induce long-lasting epigenetic changes	May alter glucocorticoid receptor sensitivity and stress-system recovery	Strong gene–environment evidence	(77)
CRH/LC-NE system	Stress and arousal regulation	Endogenous opioids can oppose CRH-related activation of locus coeruleus noradrenergic circuits	Connects opioid signalling to stress arousal rather than only reward	Mechanistic stress-system evidence	(78, 79)
Dynorphin*/*KOR	Aversive opioid-stress signalling	Dynorphin and kappa opioid receptor activity are linked to dysphoria, stress-induced seeking, and relapse	Provides a counter-system to MOR-mediated comfort and social soothing	Strong preclinical and translational support	(80–82)
*SLC6A4*	Serotonin reuptake transporter	Methylation at *SLC6A4* has been linked to family history of child abuse	May alter serotonin reuptake and long-term serotonergic tone	Direct childhood adversity evidence	(83, 87)
*TPH2*	Serotonin synthesis enzyme (CNS)	Early-life stress has been associated with *TPH2* methylation and antidepressant response	May affect central serotonin synthesis and downstream β-endorphin responses	Direct early-life stress evidence	(88)
*MAOA*	Monoamine degradation enzyme	Maltreatment-related effects depend on genotype and methylation status	May alter degradation of serotonin and related monoamines	Direct maltreatment evidence, but not serotonin-specific	(89, 90)
*HTR1A*	5-HT_1A_ receptor (inhibitory)	Promoter methylation has been reported in psychiatric disorders	Mechanistically relevant because 5-HT_1A_ activation can facilitate β-endorphin release	Mechanistic candidate; trauma-specific evidence limited	(33, 51, 91)
*HTR2A*	5-HT_2A_ receptor (excitatory)	Methylation changes have been reported in psychiatric phenotypes	May influence emotional reactivity and stress-related serotonergic signalling	Candidate evidence; trauma-specific evidence limited	(45, 92)
*OXTR*	Oxytocin receptor	Low maternal care has been associated with increased *OXTR* methylation in blood cells	May affect social buffering, attachment and stress responsivity	Direct early-care and maltreatment-related evidence	(93–96)
*OPRM1/*MOR	mu-opioid receptor (MOR)	*OPRM1* methylation has been linked to early-life adversity and response to social distress	May alter social reward, distress regulation, and opioid-mediated soothing	Direct early-adversity evidence, but limited replication	(69, 70, 97, 98)
*DRD1*	D1 dopamine receptor (excitatory)	Early-life stress alters transcriptomic patterning across reward circuitry	May affect motivational salience and indirect POMC or β-endorphin regulation	Indirect molecular evidence	(99)
*DRD2*	D2 dopamine receptor (inhibitory)	*DRD2* promoter methylation has been associated with childhood abuse exposure in clinical samples	May influence reward processing and motivational salience	Direct clinical methylation evidence	(100)
*POMC*	β-endorphin precursor	POMC promoter methylation has been examined in peripheral leukocytes in metabolic studies	May affect endogenous β-endorphin regulation, but trauma-specific evidence is limited	Mechanistic candidate; indirect evidence	(101)

Taken together, these findings support the plausibility that early adversity can affect several components of the attachment-related motivational system. The evidence is strongest for some pathways, especially stress-axis genes, *SLC6A4*, *TPH2*, *OXTR*, *OPRM1*, and *DRD2*. It is more indirect for others. The table therefore does not claim that each alteration independently causes depression. Rather, it identifies molecular points at which early adversity may disturb serotonergic, oxytocinergic, dopaminergic, opioidergic, and stress-regulatory systems.

Within the present model, the clinically relevant consequence is reduced capacity to maintain stable serotonergic and β-endorphin regulation during attachment-related stress. This remains a model-based interpretation. It is nevertheless consistent with the observation that behaviours frequently comorbid with depression, such as obesity, alcohol misuse, and substance use, can acutely increase β-endorphin levels (e.g., 16, 20, 34). These behaviours may therefore function as short-term compensatory responses to chronic opioid dysregulation rather than as completely independent primary disorders. While such compensation may transiently stabilise affective state, it does not restore attachment-related regulation and may introduce additional psychopathology.

## Therapeutic implications

If depression is conceptualised as partly related to trauma-induced disruption of attachment and serotonergic regulation, therapeutic strategies must address more than acute neurotransmitter availability. Pharmacological interventions may reduce symptoms. Their effect, however, depends on receptor availability, downstream signalling, stress-system state, and the capacity of attachment-related circuits to respond.

A pharmacological intervention may be most informative when a disturbance of the attachment drive is clinically indicated. Its benefit would likely increase if the *type* of disturbance could be specified, rather than inferred from symptoms alone. Direct assessment of epigenetic changes in central nervous tissue is not feasible in routine practice. This has led to the pragmatic use of peripheral samples as partial surrogates. They may be useful only when interpreted together with developmental history, symptom pattern, comorbidity, and treatment response.

**Table 3** summarises sample sources and assay formats that have been used to quantify epigenetic variation in candidate genes relevant to the present model.

**Table 3:** Methods to quantify epigenetic variation in candidate genes relevant to attachment related symptom patterns.

Gene	Peripheral sample source	Epigenetic readout	Ref.
*HTR1A*	Peripheral blood leukocytes	CpG DNA methylation (promoter or regulatory region)	(91)
*HTR2A*	Saliva DNA	CpG DNA methylation (targeted CpG site or short region)	(92)
*SLC6A4*	Peripheral blood leukocytes or PBMCs	CpG DNA methylation (promoter or regulatory region)	(87, 102, 103)
*TPH2*	Peripheral blood	CpG DNA methylation (targeted)	(88)
*MAOA*	Peripheral blood	DNA methylation (targeted)	(89)
*DRD2*	Peripheral blood lymphocytes	CpG DNA methylation (5 prime regulatory region)	(100)
*OXTR*	Peripheral blood leukocytes	CpG DNA methylation (CpG island or promoter)	(93–96)
*OPRM1*	Peripheral blood	Promoter DNA methylation	(97, 104)
*POMC*	Peripheral blood leukocytes	Promoter CpG methylation	(101)

The genes listed above have been used in clinical and translational studies to quantify DNA methylation changes in pathways relevant to social affiliation, stress regulation and reward processing.

Selective serotonin reuptake inhibitors (SSRIs) increase synaptic serotonin availability and may indirectly enhance β-endorphin-related signalling through serotonergic and 5-HT_1A_ -linked mechanisms (33, 49, 51). This mechanism can stabilize mood and reduce anxiety in many patients with major depressive disorder (7). It does not, however, guarantee restoration of attachment-related reward or soothing. Trauma-associated changes affecting *SLC6A4*, *HTR1A*, or broader stress regulation may limit the translation of serotonergic tone into opioid-mediated satisfaction (87, 91, 102, 103).

Adjunctive serotonergic agents with partial agonist properties at the 5-HT_1A_ receptor remain mechanistically plausible because 5-HT_1A_-related signalling may influence β-endorphin responses (33, 51). Agents acting on 5-HT_2A_ or 5-HT_2C_ pathways are more difficult to interpret, because these receptors are assigned different positions in the present model. *HTR2C* is linked mainly to tonic dopaminergic control in the general drive architecture, whereas *HTR2A* is linked more closely to phasic dopaminergic activation during attachment-related social interaction (25, 41–45). The clinical relevance of 5-HT_2A_ or 5-HT_2C_ pathways for trauma-related depression is therefore likely to depend on symptom profile and circuit state. These approaches remain symptom-oriented. They do not reverse trauma-induced molecular alterations.

Direct pharmacological manipulation of the endogenous opioid system presents stronger conceptual problems. Mu-opioid receptor activation can acutely improve affective stability, social soothing, or emotional numbing. However, clinically available agonists carry a substantial risk of dependence (105–107). Partial agonists or mixed agonist-antagonist approaches have shown antidepressant signals in selected studies, including buprenorphine/samidorphan trials, but results remain insufficient for routine trauma-related depression treatment (108–111). This supports the model’s therapeutic caution. Opioid mechanisms are relevant, but direct substitution is not a simple solution.

Kappa opioid receptor modulation is another theoretical route. Dynorphin/kappa signalling is closely linked to stress-induced dysphoria, anxiety-like behaviour, and relapse vulnerability (80–82, 112). Kappa antagonism may therefore be relevant for the aversive component of trauma-related opioid dysregulation. At present, this remains mainly experimental. It should be viewed as a counter-system to mu-opioid-mediated comfort, not as a replacement for attachment repair.

Dopaminergic approaches require similar caution. Dopamine receptors are divided into D1-like and D2-like families with distinct signalling properties (113). D1 and D2 mechanisms can influence reward learning, motivation, and POMC or β-endorphin output in preclinical systems (99, 100, 114). Yet dopaminergic modulation is unlikely to restore attachment-related satisfaction by itself. It may increase salience or activation without resolving impaired opioid-mediated soothing.

Cannabinoid CB1 receptor activation has been proposed as a more indirect means of enhancing β-endorphin signalling. Importantly, this route may bypass the serotonergic and dopaminergic steps that are otherwise required to drive β-endorphin release. Preclinical studies suggest that CB1 stimulation can activate hypothalamic POMC neurons and affect feeding-related β-endorphin release (115). Medical cannabis has also been discussed in relation to mental health outcomes, but the clinical evidence remains mixed and context-dependent (116). For this reason, cannabinoid modulation remains preliminary in the present model.

**Table 4** summarises pharmacological strategies that may modulate receptor systems affected by trauma-related epigenetic or molecular alterations. The table therefore illustrates possible mechanistic entry points rather than established biomarker-guided treatment rules.

**Table 4:** Pharmacological modulation of epigenetically altered systems.

System/receptor	Role in the model	Possible pharmacological Modulation	Potential clinical effect	Main caution	Key refs.
*HTR1A* (5-HT_1A_)	Links serotonergic tone to stress regulation and β-endorphin responses	SSRIs, or partial agonists	May improve mood, anxiety and downstream opioid-related signalling	Receptor methylation or dysfunction may limit response	(33, 49, 51, 91)
*HTR2A* (5-HT_2A_)	Supports phasic dopaminergic activation and plasticity relevant to attachment-related social interaction in **Figure 3**	Atypical antipsychotics, experimental serotonergic agents; receptor-specific approaches	May influence emotional reactivity, social salience, and trauma-related hyperarousal	Candidate component; trauma-specific evidence remains limited	(43, 45, 92)
*HTR2C* (5-HT_2C_)	Regulates tonic dopaminergic and noradrenergic output relevant to the general Freudian drive architecture in **Figure 2**	Indirect modulation through serotonergic agents; experimental receptor-specific approaches	May influence background arousal, impulse control, and drive execution	Not attachment-specific; effects are region- and state-dependent	(39, 41, 42)
*OXTR* (Oxytocin receptor)	Supports social buffering and attachment-related regulation	Intranasal oxytocin (experimental)	May improve social salience or stress buffering in selected contexts	Response may depend on context, sex, early adversity, and receptor methylation	(53, 56, 64, 71, 93–96)
β-endorphin/POMC	Endogenous opioid peptide system involved in soothing, reward, and stress regulation	Exercise, SSRIs (indirect), electroconvulsive therapy, experimental opioid modulation	May reduce anhedonia and support emotional regulation	Impaired POMC activity may limit endogenous β-endorphin regulation	(8, 10, 12, 19, 33, 49, 51, 101)
*OPRM1*/MOR	Mediates social reward, affective comfort, analgesia, and opioid reinforcement	Buprenorphine; buprenorphine/samidorphan; naltrexone in selected contexts	May improve affective stability or reward sensitivity in selected cases	Direct agonism is limited by dependence risk; receptor availability may be altered in depression/anxiety	(69, 70, 97, 98, 104, 111)
dynorphin/KOR	Aversive opioid-stress system linked to dysphoria and stress-induced seeking	KOR antagonists, mainly experimental	May reduce stress-induced dysphoria and relapse vulnerability	Translational evidence remains incomplete	(80–82, 112)
*DRD1* (D1 receptor)	May contribute to POMC or β-endorphin output in preclinical systems	No D1-selective drugs are clinically established for depression	Theoretical mood and cognitive activation	Evidence for β-endorphin regulation is indirect and mainly preclinical	(99, 113, 114)
*DRD2* (D2 receptor)	D2 regulates reward processing, motivational salience, and inhibitory dopaminergic control	Atypical antipsychotics for D2-related pathways	May reduce anhedonia or improve reward responsive-ness in some patients	Effects on β-endorphin tone remain variable and context dependent	(100, 113, 114)
CB1 receptor	Cannabinoid receptor Interacts with hypothalamic POMC neurons and feeding-related reward	Dronabinol or related Dronabinol or related cannabinoid modulation, experimental in this context	May affect appetite, anxiety, and mood-related symptoms	Psychiatric effects are mixed; evidence remains preliminary	(115, 116)

Given these pharmacological constraints, psychotherapeutic interventions remain central rather than peripheral. This follows directly from the model. If trauma-related depression involves impaired attachment-related motivation, then treatment cannot be limited to preserving serotonergic tone or modulating receptor systems. The damaged function is relational and motivational. It must therefore be approached in a relational and motivational setting.

Medication can reduce symptoms and may help to stabilise serotonergic signalling. Yet relapse remains common. Meta-analytic evidence indicates that relapse after antidepressant discontinuation is frequent, and relapse also remains clinically relevant during continued treatment. In a large primary-care trial, 39% of patients who continued antidepressants relapsed within 52 weeks, compared with 56% after discontinuation. Recent meta-analytic data also indicate relapse rates of about 35% after 6 months and 45% after 12 months following discontinuation (117, 118). These figures do not argue against medication. They show its limits.

Psychotherapy addresses a different level of the problem. Sequential meta-analytic evidence suggests that adding psychotherapy after response to acute pharmacotherapy reduces relapse or recurrence in major depressive disorder (119). Psychological interventions added to antidepressants also reduce relapse risk compared with antidepressants alone (120). This supports the clinical assumption that pharmacological improvement and motivational recovery are not identical. A patient may become less symptomatic without having restored attachment-related safety, social trust, or reward responsiveness.

This point is especially relevant for patients with attachment disturbance. Trauma-focused cognitive behavioural therapy and attachment-based psychotherapies address traumatic expectations, relational insecurity, affect regulation, and avoidance (121, 122). They do not simply “talk about” trauma. They provide repeated corrective experiences in which affect can be tolerated, meanings can be reorganised, and interpersonal expectations can be tested under safer conditions.

Group-based approaches deserve particular attention. The present model assumes that attachment-related regulation is not restored by insight alone. It requires repeated social experience. Group therapy can provide mirroring, interpersonal feedback, shared affect regulation, and graded re-entry into social relatedness. In survivors of childhood sexual abuse, a randomized trial found that group psychotherapy reduced trauma-related symptoms compared with waitlist, and adequate-dose analyses also showed reductions in depression, dissociation, and sexual concerns (123). These findings are clinically important because they concern precisely the type of relational trauma in which attachment, shame, social avoidance, and opioid-mediated comfort may be disturbed.

Behavioural activation is also relevant. It does not directly repair attachment, but it counters withdrawal and increases contact with rewarding activities (124). Within the present model, this may help to increase opportunities for endogenous reward and social reinforcement. The same logic applies to body-oriented and trauma-sensitive approaches, although the evidence base is more heterogeneous. They may support affect tolerance, body ownership, and stress regulation, but they should not be presented as direct proof of β-endorphin normalisation.

Taken together, these interventions fit the model because they act where medication is weakest. Pharmacological treatment may support neurochemical availability. Psychotherapy may provide the interpersonal and behavioural conditions in which attachment-related motivation can be rebuilt. The model therefore predicts that the most stable outcomes should occur when pharmacological stabilisation is combined with interventions that address attachment, avoidance, shame, and social reward.

## Neurochemical scope and boundary conditions of the model

The revised model is not intended to reduce trauma-related depression to a single pathway. Childhood trauma has been linked to altered HPA-axis activity, allostatic load, and stress-related neurodevelopmental changes (125–130). These findings support the view that early adversity changes stress responsivity rather than simply changing mood. They also explain why trauma-related depression may overlap with anxiety, substance use, somatic symptoms, and interpersonal dysregulation. Large epidemiological studies further show that childhood adversities are transdiagnostic risk factors, not disorder-specific causes (131–133).

Attachment-related regulation also requires a broader neurochemical frame. Human attachment depends on coordinated activity across oxytocinergic, dopaminergic, opioidergic, autonomic, and sensory systems (134, 135). Social buffering can reduce stress responses, and oxytocin contributes to this process in both animal and human studies (136, 137). Affective touch is especially relevant here. It provides a direct sensory route through which caregiving, safety, and social reward can be encoded (138, 139). This supports the model’s emphasis on physical and emotional aspects of early attachment.

The opioid part of the model is also broader than β-endorphin concentration alone. Social attachment has long been linked to endogenous opioid mechanisms (140). More recent work suggests that mu-opioid signalling contributes to social motivation, social connection, and the affective value of interpersonal contact (141–144). This is compatible with the present interpretation of β-endorphin-mediated satisfaction, but it also adds caution. Receptor availability, social context, and state-dependent responsivity may be as important as peptide level.

The kappa-opioid system provides a necessary counterweight. KOR signalling is linked to stress, aversion, negative affect, and drug seeking (80–82, 112, 145–148). This prevents the model from becoming a simple comfort-deficit account. Trauma-related depression may involve impaired mu-opioid-mediated soothing and increased kappa-related dysphoria. In some patients, early-life stress may also alter opioid sensitivity and increase vulnerability to later opioid misuse (149, 150).

Serotonergic and dopaminergic systems add further complexity. Serotonin and dopamine interact in opponent and cooperative ways, depending on receptor subtype, brain region, and behavioural state (151–153). Dopamine supports incentive salience, reward learning, and effortful activation, whereas opioids contribute more directly to hedonic value and social comfort (29, 30, 154–156). Learned helplessness research also links stress controllability, *dorsal raphe* serotonin, and motivational shutdown (157). This is relevant for trauma-related depression, where passivity and withdrawal may reflect altered motivational control rather than simple sadness.

These findings do not prove the updated drive model. They define its biological boundary conditions. The model is strongest where attachment disruption, stress dysregulation, altered reward responsivity, and compensatory behaviour occur together. It is weaker when depression arises primarily from inflammatory, metabolic, neurological, or purely situational pathways. This limitation is important, but it does not undermine the model. It defines the clinical phenotype to which the model should be applied. This view is also compatible with the proposal that maltreatment-related psychopathology may include ecophenotypic variants with partly distinct neurobiological profiles (158). Broader reviews of child abuse, neglect, and youth psychopathology likewise support a transdiagnostic, developmentally sensitive interpretation rather than a single-disorder model (159, 160).

## Discussion

The present model proposes that a subset of depressive disorders can be understood as a consequence of trauma-related disruption of attachment and its neurobiological regulation. Within this model, serotonergic dysregulation and altered β-endorphin signalling are not treated as isolated abnormalities. They are viewed as interconnected outcomes of impaired motivational, stress-regulatory, and attachment-related systems (36–45, 53–56, 75–84).

A central implication is that depression should not be conceptualised solely as a disorder of neurotransmitter deficiency. Serotonergic signalling is important, but its role is regulatory rather than exclusive. It interacts with dopaminergic, oxytocinergic, opioidergic, and HPA-axis mechanisms (38–45, 53–56, 75–82). This broader view is necessary because early adversity does not affect a single transmitter system. It can alter stress responsivity, social buffering, reward learning, and the capacity for interpersonal safety (76, 77, 83–87, 93–98).

The distinction between dopamine and endogenous opioids remains central to the model. Dopamine supports salience, learning, approach behaviour, and searching. It does not provide final satisfaction by itself (29, 30, 46–48). β-Endorphin and mu-opioid signalling are more closely related to soothing, reward value, affective comfort, and the termination of motivational pressure (9–12, 49–52, 69, 70, 97, 98, 105, 106). In the present manuscript, satisfaction is used primarily in the Freudian sense of drive fulfilment. For academic clarity, however, related functional-neurochemical accounts have also associated MOR activity and opioid-receptor systems with emotional valence, dispositional satisfaction, endurance-related regulation, and attachment-related maternal behaviour (161–164). This distinction helps preserve the Freudian idea of drive fulfilment without reducing it to general behavioural activation (20–25, 31, 32, 34).

The opioid component must also be broadened beyond a simple β-endorphin deficit. Mu-opioid mechanisms are relevant for social comfort and reward, but dynorphin and kappa opioid receptor signalling are more closely linked to stress-related dysphoria and compulsive seeking (80–82, 105–112). Trauma-related depression may therefore involve both impaired mu-opioid-mediated soothing and increased aversive opioid-stress signalling. This makes the model less linear and more compatible with current opioid neuroscience.

The association between altered opioid tone and behaviours such as substance use, overeating, or compulsive activity gains particular relevance in this context. Such behaviours can acutely increase endogenous opioid activity and may function as short-term compensatory responses to chronic dysregulation (15, 19, 72–74, 107). They should not be treated as proof of the model. They are better interpreted as clinical phenomena that the model can help to organise.

From a therapeutic perspective, these considerations may explain why pharmacological approaches alone often produce incomplete responses in trauma-related depression. SSRIs may preserve serotonergic tone and indirectly facilitate β-endorphin-related signalling (33, 49–51). Yet their effect depends on receptor availability, downstream signalling, stress-system state, and attachment-related circuit responsivity (87, 91, 97, 98, 102–104). This may help explain why patients with prominent early adversity or attachment disturbance can show partial, unstable, or delayed responses.

The updated drive model does not claim universal applicability to all depressive disorders. Other forms of depression may arise from different biological or psychosocial pathways, including genetic vulnerability, inflammatory processes, metabolic factors, pain states, or medical illness (7, 83, 84). The model is most relevant to trauma-related presentations in which attachment disruption, interpersonal avoidance, anhedonia, and compensatory behaviours are clinically prominent (57–74, 117–124).

Finally, this model reframes selected aspects of Freudian drive theory in a manner compatible with contemporary neuroscience. Freud lacked access to neurochemical concepts. His insistence on a limited number of drives and a shared internal aim can nevertheless be reconsidered in light of motivational neuroscience and attachment-related neurobiology (21–25, 29–32, 46–56, 61–70). The present reinterpretation does not require acceptance of classical psychoanalytic assumptions. It treats them as historically grounded hypotheses that may be refined through current neurobiology.

## Limitations

Several limitations of the present framework should be acknowledged.

First, the model is selective. It focuses on serotonergic regulation, β-endorphin-related signalling, attachment, and stress-related opioid mechanisms. Depression is broader than this. Glutamatergic neurotransmission, inflammation, immune activation, neurogenesis, metabolic state, pain, sleep, and endocrine mechanisms are not fully integrated here.

Second, childhood trauma is considered a relevant risk factor only for a specific subtype of depression. It is not presented as a necessary or sufficient cause. Many depressive disorders arise without identifiable early adversity. Conversely, childhood trauma can lead to anxiety disorders, personality pathology, substance use, post-traumatic stress disorder, or no diagnosable disorder.

Third, much of the evidence linking trauma to epigenetic modification of serotonergic, oxytocinergic, dopaminergic, opioidergic, and stress-related genes remains correlational. Human studies differ in tissue source, adversity measurement, timing, sex distribution, and clinical phenotype. Causal inference therefore remains limited.

Fourth, peripheral biomarkers are imperfect. Peripheral DNA methylation may be clinically useful in some contexts, but it cannot be equated with central gene expression. The same caution applies to β-endorphin. Peripheral peptide levels do not reliably reflect central opioid tone, receptor availability, or phasic opioid responses during social interaction.

Fifth, the model often refers to β-endorphin tone in a general sense. This should not be understood as a single static quantity. In dopamine research, tonic and phasic signalling have long been distinguished, and this distinction is relevant for craving, reward responsivity, and addiction. A similar caution is useful for endogenous opioids. A patient may show altered basal opioid tone while still having impaired phasic opioid responses during attachment, stress, or reward.

Within the updated drive model, this distinction may also help to clarify why the general drive architecture and the attachment drive are not neurobiologically identical. Earlier work proposed that physiological Freudian drives may be linked to serotonergic regulation of tonic dopaminergic activity, whereas the attachment system may rely more strongly on phasic dopaminergic responses during social interaction (25). This interpretation remains model-based and requires direct empirical testing.

Sixth, the therapeutic implications are inferential. No clinically established method currently restores endogenous β-endorphin regulation in a trauma-specific and receptor-specific manner. Available pharmacological agents act indirectly or carry substantial risks. Psychotherapy may support attachment-related regulation, but direct evidence that it normalises β-endorphin or mu-opioid signalling in humans remains limited.

Finally, constructs such as drives and internal aims are not standard categories in modern neurobiology. Their translation into receptor-level, circuit-level, and interpersonal mechanisms remains partly theoretical. The model should therefore be read as a testable synthesis, not as a completed explanation.
